# Sodium-Glucose Cotransporter 2 Inhibitor Therapy in Different Scenarios of Heart Failure: An Overview of the Current Literature

**DOI:** 10.3390/ijms252111458

**Published:** 2024-10-25

**Authors:** Silvia Prosperi, Andrea D’Amato, Aurora Labbro Francia, Sara Monosilio, Claudia Cestiè, Stefanie Marek Iannucci, Lucrezia Netti, Danilo Angotti, Domenico Filomena, Marco Valerio Mariani, Vincenzo Myftari, Rosanna Germanò, Sara Cimino, Massimo Mancone, Roberto Badagliacca, Viviana Maestrini, Paolo Severino, Carmine Dario Vizza

**Affiliations:** 1 Department of Clinical, Internal, Anesthesiology and Cardiovascular Sciences, Sapienza University of Rome, Viale del Policlinico 155, 00161 Rome, Italy; silviapro@outlook.it (S.P.); auro1298@gmail.com (A.L.F.); sara.monosilio@gmail.com (S.M.); claudia.cestie@gmail.com (C.C.); stefanie.marekiannucci@gmail.com (S.M.I.); lucrezia.netti@gmail.com (L.N.); danilo.angotti95@gmail.com (D.A.); domenico.filom@gmail.com (D.F.); marcoval.mariani@gmail.com (M.V.M.); vincenzo.myftari@gmail.com (V.M.); rosanna.germano@gmail.com (R.G.); cimins.87@gmail.com (S.C.); massimo.mancone@uniroma1.it (M.M.); roberto.badagliacca@uniroma1.it (R.B.); viviana.maestrini@uniroma1.it (V.M.); paolo.severino@uniroma1.it (P.S.); dario.vizza@uniroma1.it (C.D.V.); 2 Department of Cardiology, Ospedale Fabrizio Spaziani, 03100 Frosinone, Italy

**Keywords:** sodium-glucose cotransporter 2 inhibitors, heart failure, coronary artery disease, amyloid cardiomyopathy, cardiotoxicity, Takotsubo syndrome, valvular heart disease

## Abstract

Heart failure (HF) is a complex syndrome that requires tailored and patient-centered treatment. Sodium-glucose cotransporter 2 inhibitors (SGLT2is) constitute one of the four pillars of the medical treatment of HF. However, the 2023 ESC guidelines treat HF as a single entity without making clear distinctions in phenotypes according to etiology. This creates a “gap in knowledge”, causing much debate about the applicability of these drugs in peculiar clinical settings that are etiological and/or predisposing clinical conditions for HF. Furthermore, considering the variety of etiologies and different pathophysiological backgrounds of HF, one might question whether the use of SGLT2is is equally beneficial in all types of HF and whether certain drug-related properties may be exploited in different contexts. For example, SGLT2is can improve the metabolic and inflammatory state, which is fundamental in ischemic heart disease. Anti-inflammatory power can also play a paramount role in myocarditis or cardiotoxicity, while improving the congestive state and reducing filling pressure may be even more fundamental in restrictive heart disease or advanced heart disease. This review aims to gather the evidence currently present in the literature concerning the advantages or the disadvantages of using these drugs in these particular clinical settings, with the goal being an optimized and highly personalized treatment for HF.

## 1. Introduction

Sodium-glucose cotransporter 2 inhibitors (SGLT2is) constitute one of the four pillars of the medical treatment of heart failure (HF), regardless of ejection fraction and diabetic status, as stressed by the latest update of the 2023 ESC guidelines [1,2,3,4].

All the major HF trials, including the DAPA-HF and EMPEROR-Reduced, have demonstrated that the use of SGLT2is leads to an improvement in symptoms and a reduction in mortality early on, respectively 28 and 12 days from treatment initiation [5,6]. It is therefore not surprising that the implementation of SGLT2is is recommended early on when initiating the guideline-directed medical treatment (GDMT) of HF. That way, the cardioprotective and nephroprotective effects of SGLT2is can unfold early [7,8,9].

Considering the variety of etiologies and different pathophysiological backgrounds of HF, one might question whether the use of SGLT2is is equally beneficial in all types of HF and whether certain drug-related properties may be exploited in specific contexts.

Certain clinical settings may benefit more than others from specific properties, highlighting the importance of tailored, patient-centered treatment in today’s medical landscape. The objective of this review is to provide a comprehensive overview of the current use of SGLT2is across various HF subgroups based on different etiologies. Special emphasis will be placed on scenarios not officially addressed in the guidelines or major studies but frequently encountered in clinical practice.

**Figure 1:**
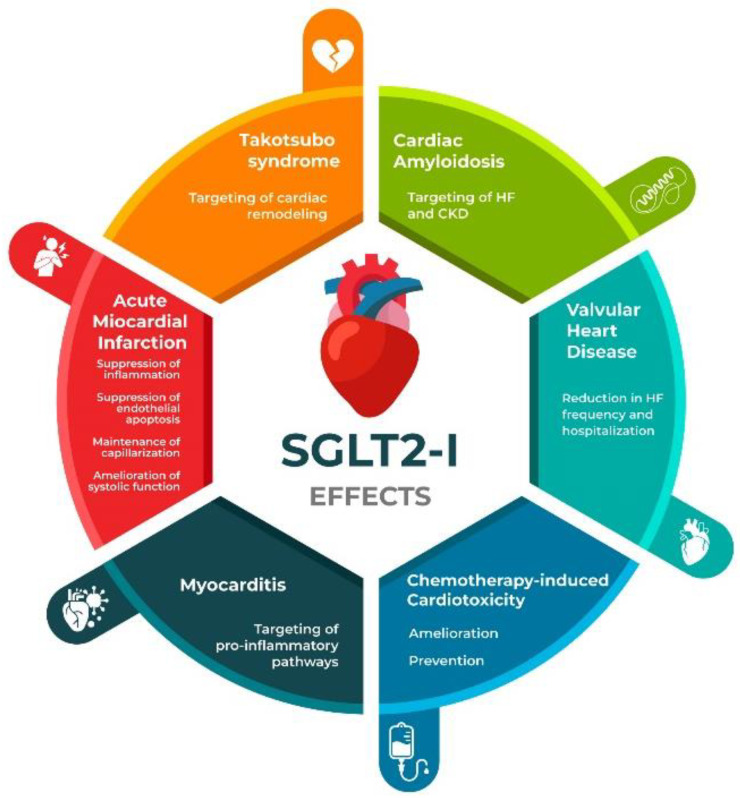
Summary of SGLT2is’ actions on different pathophysiological backgrounds of heart failure. HF: heart failure; CKD: chronic kidney disease.

Figure 1 summarizes SGLT2is’ potential actions on different pathophysiological grounds of HF.

## 2. SGLT2is and Coronary Artery Disease: From Pathophysiological Mechanisms to Clinical Applications

Myocardial infarction (MI) is the leading cause of HF development, accounting for 15% of symptomatic HF with or without reduced ejection fraction (HFpEF and HFrEF respectevely). Therefore, one should question whether the beneficial effects of SGLT2is on HF may also prove protective in the setting of MI, possibly preventing the development of HF itself [10].

### 2.1. SGLT2is and Coronary Artery Disease: Mechanisms

SGLT2is can affect both acute (<6 h) and delayed mechanisms occurring during ischemic myocardial damage. In the acute setting, there is a reduction in intracellular sodium (Na^+^) and calcium (Ca^2+^), a reduction in the phosphorylation of adenosine monophosphate-activated kinase (AMPK) alpha and oxidative stress, and the inhibition of the sodium (Na^+^)/hydrogen (H^+^) exchanger (NHE) [11]. During the delayed phase, there is a reduction in calcium/calmodulin-dependent protein kinase II (CaMKII) activity, inflammation, and oxidative stress, while there is an upregulation of glucose uptake and signal transducer and activator of transcription 3 (STAT3) [11]. These mechanisms may have a pivotal role in the reduction of infarct size in the corresponding phase. The main cell types that have been found to be responsible for the cardioprotective effects displayed by SGLT2is are cardiomyocytes, fibroblasts, and endothelial cells [11].

Significant knowledge remains to be gained about the pleiotropic effects of SGLT2is, as ongoing studies investigate various targeted pathways. For example, Nakao et al. developed a left ventricular (LV) pressure overload model in mice through transverse aortic constriction to assess Empagliflozin’s effects on HF [12]. The administration of Empagliflozin improved pressure overload-induced systolic dysfunction and increased citrulline levels in heart tissue while reducing arginine levels, indicating the enhanced metabolism of arginine to citrulline and nitric oxide (NO) [12]. Transcriptome analysis suggested that there could be involvement of the insulin/protein kinase B (AKT) pathway, leading to the activation of nitric oxide (NO) production through endothelial NO synthase (eNOS) phosphorylation. Empagliflozin treatment was also associated with significant improvement in the histological examination of capillary rarefaction and endothelial apoptosis after transverse aortic constriction. Such an improvement possibly led to increased expression of phospho-eNOS and NO production in cardiac endothelial cells [12]. Hence, Empagliflozin activates the AKT/eNOS/NO pathway, which supports the suppression of endothelial apoptosis, maintains capillarization, and ameliorates systolic dysfunction in a setting of LV pressure overload [12].

### 2.2. SGLT2is and Coronary Artery Disease: Literature Overview

Several studies have been carried out, aiming to study the potential beneficial effects of SGLT2is on coronary artery disease (CAD). For instance, one study included 97 participants with type 2 diabetes mellitus (T2DM) and coronary artery disease; 49 were randomized to being treated with Empagliflozin, and 48 were randomized to receive a placebo. Empagliflozin has been shown to significantly reduce LV mass indexed to body surface area after 6 months of treatment [13]. However, such a study, apart from being limited in terms of the small number of participants, is also limited to a specific subset of CAD-affected patients, which is, patients affected by T2DM.

MI is a much less explored field, possibly because this subset of patients has been relatively understudied in trials to date. One study has demonstrated that Empagliflozin suppresses cardiomyocyte autosis (an autophagic, non-apoptotic, and non-necrotic form of cell death) by inhibiting the activity of the cardiomyocyte Na^+^/H^+^ exchanger 1 (NHE1), hence conferring cardioprotective effects. It significantly reduced infarct size and myocardial fibrosis, therefore resulting in improved cardiac function and survival [14].

Experimental models of both diabetic and nondiabetic acute MI demonstrate that SGLT2is confer several benefits, including reduced neurohormonal activation, cardiomyocyte injury, and reperfusion injury. Additionally, SGLT2is may enhance endothelial function and vasodilation, improve myocardial energy metabolism, preserve contractility, and attenuate oxidative pathways to enhance coronary blood flow [15]. SGLT2is may display other beneficial effects in a high-risk post-MI population, including reduction in afterload and preload, glucose control, and weight loss by means of glycosuria and natriuresis. Additionally, their nephroprotective effects may further improve plasma volume balance and myocardial oxygen delivery. However, it is to be acknowledged that it is yet to be discovered whether treatment with SGLT2is is effective and safe early post-MI [15]. Indeed, on the one hand, some patients with acute MI may share features of conditions for which SGLT2is are currently indicated; on the other hand, however, this does not mean that they will necessarily prove to be beneficial in such a setting. Hence, further studies are needed to clarify the latter and to establish whether the pleiotropic effects of SGLT2is may be safe and beneficial in the early post-acute MI period [15].

Dapagliflozin has been investigated regarding myocardial ischemia/reperfusion injury (MIRI)-related ferroptosis. It is well known that timely reperfusion is the key to rescuing ischemic myocardial tissue while also preventing the occurrence of HF. However, reperfusion itself may induce some degree of myocardial damage, namely, MIRI. The mechanisms underlying MIRI are numerous and diverse [16]. Among these, there is ferroptosis, which is a type of programmed cell death with intracellular iron overload, which then leads to the overproduction of reactive oxygen species (ROS), resulting in lipid peroxidation and ferroptosis [16]. Chen W et al. demonstrated in rat models that Dapagliflozin was capable of significantly improving myocardial injury, reperfusion arrhythmia, and cardiac function, as demonstrated by ameliorated cardiac biomarkers, such as cardiac troponin T (cTnT) and B-type natriuretic peptide (BNP), and by improvements in terms of ST-segment elevation. Indeed, Dapagliflozin attenuated oxidative stress, iron overload, lipid peroxidation, and ferroptosis [12].

The administration of Empagliflozin has shown acute functional protective effects, such as the improvement of cardiac performance during ischemic episodes, similar to Dapagliflozin. Protective mechanisms that have been proposed include NHE inhibition, the reduction of cytosolic levels of Na^+^ and Ca^2+^, the inhibition of oxidative stress, the activation of STAT3, and a reduction in cardiac inflammation [17,18].

Further evidence on Empagliflozin has been provided by the EMMY trial, a multicenter, double-blind study, in which patients with acute MI and a large creatine kinase elevation (>800 IU/L) were randomly assigned to either receive Empagliflozin 10 mg or a placebo daily, within 72 h of percutaneous coronary intervention (PCI) [10]. Patients treated with Empagliflozin had a significantly larger NT-proBNP decrease over 26 weeks, together with significant improvement in echocardiographic function and structural parameters, when compared to patients treated with a placebo. A limitation of the study, however, is the fact that its sample size was insufficient to power it for hard clinical endpoints [10].

A post hoc analysis investigated whether SGLT2is may improve the metabolism of ketone bodies within the myocardium. Therefore, changes in serum levels of beta-hydroxybutyrate (3-βOHB) were analyzed among patients who received either Empagliflozin or conventional treatment of MI for a period of 26 weeks post-MI [19]. Baseline and repeated measurements of 3-βOHB with cardiac parameters were studied. Cardiac parameters included NT-proBNP, left ventricular ejection fraction (LVEF), LV end-systolic volume (LVESV), LV end-diastolic volume (LVEDV), and left ventricular filling pressure (E/e’ ratio). The study revealed that the mean 3-βOHB levels increased from baseline to 6 weeks and 26 weeks in the Empagliflozin group compared to the placebo group, in which a significant decline could be appreciated at 26 weeks [19]. Higher baseline 3-βOHB levels were negatively correlated with cardiac functional and structural markers, while an increase in these levels over time improved these markers. These results highlight the importance of studying the metabolism of ketone bodies following an acute MI over a longer period [19]. Moreover, the study showed that the positive effect exerted by 3-βOHB on cardiac markers was more evident in the later stages and only in the SGLT2i group, possibly suggesting that SGLT2is may mediate these beneficial effects. However, these are preliminary findings, and more research is needed to validate these hypotheses [19].

Regarding Empagliflozin, it has been shown to reduce, in patients with acute MI, the risk of first and total HF hospitalizations across the range of LV ejection fraction values, irrespective of the presence of congestion [20].

Conversely, in a recent double-blind, randomized, placebo-controlled trial of patients hospitalized for acute MI and at risk of HF, Empagliflozin did not significantly reduce HF hospitalization risk of death from any cause when compared to a placebo [21].

The EMPACT-MI trial, a double-blind, placebo-controlled, randomized trial, included patients hospitalized for acute MI and at risk of developing HF based on either newly developed LVEF < 45% or signs or symptoms of congestion. In this trial, Empagliflozin did not reduce the composite of HF hospitalizations and death from all causes compared to a placebo [22,23]. However, on further analysis of the study, the risk for first and total HF hospitalizations was significantly lower in the Empagliflozin group compared to the placebo group. Moreover, this study demonstrated that the need for the new use of renin–angiotensin modulators, diuretics, or mineralocorticoid receptor antagonists after hospital discharge was lower in patients treated with Empagliflozin rather than with a placebo [23].

Also, other studies have confirmed that, in acute MI patients, the SGLT2i-treated groups were found to experience fewer hospitalizations for any cause [24].

An additional study conducted in the acute MI setting investigated in-hospital and long-term prognosis in patients affected by T2DM, divided into SGLT2i users and non-SGLT2i-users. The primary endpoint was a composite of cardiovascular (CV) death, recurrent acute MI, and HF hospitalization [25]. In patients with T2DM who underwent acute MI, the use of SGLT2i was associated with a lower risk of adverse CV events during index hospitalization and long-term follow-up compared with patients treated with other oral antidiabetic drugs [25]. However, it should be acknowledged that there are certain study limitations. First, the sample size was powered to evaluate only a “class effect” but not the “doses effect”. Moreover, the fact that the study is observational is another limitation. In addition, patients revascularized using a coronary artery bypass graft (CABG), insulin treatment, and/or glomerular filtration rate (GFR) < 30 mL/min were excluded [25].

In the setting of acute MI in patients affected by T2DM, patients treated with SGLT2is had significantly lower inflammatory responses and smaller infarct sizes than those receiving other oral antidiabetic drugs (OADs), independent of glucose control [25]. The study focused on the glycemic-dependent and glycemic-independent effects of SGT2is. Regarding the glycemic-dependent effects, the study showed how stress hyperglycemia was more frequently observed in patients treated with other OADs compared to those treated with SGLT2is [26].

Considering the tight connection between stress hyperglycemia, infarct size, and inflammatory burden in the setting of acute MI, according to the results of the study, part of the anti-inflammatory effects displayed by SGLT2is may be due to the stricter control of stress hyperglycemia [26]. Concerning the glycemic-independent effect, the study showed how an SGLT2i was a significant predictor of reduced inflammatory response, irrespective of admission hyperglycemia. These findings support the hypothesis that the cardioprotective properties of SGLT2is go beyond glucose-lowering pathways [20]. This is further demonstrated by the fact that study participants who were treated with other, non-SGLT2i OADs exhibited an amplified inflammatory status, as proven by their increased levels of inflammatory biomarkers, such as neutrophil count, neutrophil-to-lymphocyte ratio (NLR), platelet-to-lymphocyte ratio (PRL), and C-reactive protein (CRP). Inflammation largely contributes to expanding infarct size, and inflammatory biomarkers have a correlation with acute MI prognosis. Such observations further furnish insights into the possible cardioprotective effects that SGLT2is may exhibit in the acute MI setting [26].

## 3. Exploiting SGLT2i Properties in Amyloid Cardiomyopathy

Amyloidosis is determined by the deposition of insoluble misfolded protein aggregates in several different organs, compromising their physiological functions. The two most frequent subtypes of amyloidosis are transthyretin amyloidosis (ATTR) and light chain amyloidosis (AL), which are characterized by amyloid fibrils consisting of transthyretin and light chains, respectively. ATTR may further be subclassified into hereditary transthyretin amyloidosis (ATTRv) and wild-type transthyretin amyloidosis (ATTRwt).

In both ATTR and AL, cardiac involvement is the most common manifestation, and most patients already show HF symptoms by the time of diagnosis. Indeed, data suggest that cardiac involvement is present in up to 50% of patients with AL and that ATTRwt may be the cause of up to 30% of HFpEF in patients above 75 years of age [27].

As previously stated, several other organs are affected by amyloidosis, as well, including, notably, the kidneys. Renal involvement is due to glomerular amyloid deposition, leading to proteinuria (mostly constituted by albuminuria) and tubular atrophy with interstitial fibrosis. Given the chronic decline of cardiac and renal function over time, in the setting of amyloidosis, the use of SGLT2is and their cardio-renal protective effects, have been investigated. This is of relevance since survival and quality of life, among this patient population, are strictly dependent on the functionality of the involved organs [28].

### SGLT2is: Promising Therapeutic Options for Amyloid Cardiomyopathy

One study retrospectively analyzed 79 patients with either ATTR or AL who were receiving either Dapagliflozin or Empagliflozin, with only 5% reporting adverse events, all of them being urinary tract infections; only two patients had to stop treatment [28]. As expected and already demonstrated in large prospective trials, a slight decrease in glomerular filtration was observed. Similarly, a slight decrease in serum sodium levels was also present, while serum glucose levels remained unchanged. A small but significant increase in hemoglobin levels was appreciated, possibly due to the hemoconcentration favored by the osmotic diuretic effect displayed by SGLT2is, leading to improved volume management. The E/e’ ratio decreased, as did pulmonary artery pressure [28]. As cardiac amyloidosis is a progressive condition, it is not surprising that an increase in high-sensitivity troponin, a decrease in LV stroke volume and cardiac output, and an increase in right ventricular thickness were recorded. Possibly, these changes were mostly due to the natural progression of the disease, inevitably leading to worsening cardiac conditions. However, the ameliorations in the volume status could be reasonably connected to SGLT2i treatment [29]. Given the above, SGLT2is seem to be beneficial in cardiac amyloidosis, as also evidenced by larger trials. Considering their importance in the HF setting, this study further suggests that their use should be encouraged in cardiac amyloidosis [28].

The use of SGLT2is in the setting of transthyretin amyloid cardiomyopathy (ATTR-CM) was also investigated in another study in terms of tolerability, clinical outcomes, and changes in NT-proBNP levels and GFR. It included 34 patients, 17 of which underwent Dapagliflozin treatment [30]. At the 3-month follow-up, most ATTR-CM patients in the Dapagliflozin treatment group had reduced NT-proBNP levels compared to the control group. Other parameters remained stable, and no adverse events were reported [30]. Moreover, after Dapagliflozin or Empagliflozin administration, despite a slight initial (and expected) drop in eGFR, the New York Heart Association (NYHA) functional class, cardiac and hepatic function, and the 6 min walking test remained stable, with no major adverse events [30]. Since HF and chronic kidney disease (CKD) are common complications of amyloidosis, SGLT2is may prove beneficial in this setting [30].

In ATTR-CM patients, SGLT2i treatment was associated with lower all-cause mortality, cardiovascular mortality, HF hospitalization, and the composite outcome of cardiovascular mortality and HF hospitalization [31]. The use of SGLT2is in ATTR-CM was well tolerated and associated with beneficial effects in terms of the above-mentioned clinical outcomes [31].

A limitation that applies to all these studies is the fact that they analyzed small study populations, hence the need for studies with larger populations to be carried out.

## 4. SGLT2is as a Potential Weapon Against Cardiotoxicity

Several studies have investigated the role of SGLT2is in the cardio-oncological setting. One of them evaluated the prognostic value of SGLT2is on all-cause mortality and cardiotoxicity among patients treated with immune checkpoint inhibitors (ICIs). The study included patients diagnosed with cancer and T2DM and treated with ICIs. The results showed that SGLT2i treatment was associated with lower all-cause mortality; however, regarding its effects on cardiotoxicity, further studies are needed [32].

The cardioprotective effects of Dapagliflozin regarding the cardiotoxicity of Doxorubicin in animal models were investigated, revealing that Doxorubicin + Dapagliflozin was associated with a more contained reduction in EF when compared to treatment with Doxorubicin alone [33]. Similarly, those treated with Doxorubicin + Dapagliflozin had less QRS prolongation and QT duration increase compared with those treated with Doxorubicin only. Moreover, while changes in sarcomyolysis, necrosis, and inflammatory cell infiltration were shown to be significant in the Doxorubicin-only group, they were instead minimal in the Doxorubicin + Dapagliflozin group. These promising results indicate that Dapagliflozin may reduce the cardiotoxic effects of Doxorubicin [33].

Also, Empagliflozin showed positive effects on Doxorubicin-induced acute cardiotoxicity, showing to be associated with a significant amelioration in terms of left ventricular end-diastolic volume and left ventricular end-systolic volume, QTc interval, infiltrative cell proliferation, karyolysis, and karyorrhexis ratios [34].

The effects of Empagliflozin on Doxorubicin-induced cardiotoxicity were also investigated in another study on mouse models. The study demonstrated that EF tended to decrease less in mice treated with Doxorubicin + Empagliflozin compared to mice treated with Doxorubicin only. Moreover, the hearts of mice treated with Doxorubicin + Empagliflozin were affected by a lower degree of myocardial fibrosis [35].

The use of SGLT2is during anthracycline-based chemotherapy in patients affected by T2DM led to improvements in terms of clinical outcomes compared with T2DM patients being treated with other, non-SGLT2i antidiabetic agents [36]. The clinical outcomes investigated were HF hospitalization, acute MI, ischemic stroke, and the composite outcome given by all the previous outcomes altogether. This study showed that despite T2DM being a concurrent risk factor for CV disease and anthracycline-induced cardiotoxicity, patients affected by T2DM undergoing treatment with SGLT2is and being treated with anthracycline-based chemotherapy had a lower incidence of ischemic stroke compared to those without T2DM. Moreover, HF hospitalization was shown to be lower in T2DM patients treated with SGLT2is [36]. This study does, however, have certain limitations. For instance, no laboratory data were provided, nor was demographic information, such as, for example, cancer staging. Additionally, certain patients were excluded, such as those with previous CV diseases; those with highly uncontrolled hyperglycemia; and those with vulnerabilities, such as metastatic cancers. This exclusion was aimed at minimizing the influence of unexpected confounding factors; however, on the other hand, it may have resulted in fewer CV events. Furthermore, the study analyzed the use of SGLT2is in AC-treated patients for only four months; hence, long-term use has not been evaluated in this study. Nevertheless, these results suggest that SGLT2is may protect against anthracycline-induced cardiotoxicity [36]. This is of particular interest, given the fact that a recent meta-analysis has shown that diabetes mellitus is a significant risk factor for anthracycline-related cardiotoxicity [37].

Given the significant number of patients undergoing chemotherapy and the strong association between chemotherapy and cardiac dysfunction and HF, the scientific interest in the field is constantly growing.

A recent retrospective cohort analysis included patients with T2DM, cancer, and exposure to potentially cardiotoxic antineoplastic drugs, with a following diagnosis of cardiomyopathy or HF. Patients treated with SGLT2is, in addition to standard guideline-directed medical therapy, carried a lower risk for acute HF exacerbation and all-cause mortality [32]. Moreover, other events, such as all-cause hospitalizations or emergency department admissions, atrial fibrillation/flutter, acute kidney injury, and the need for renal replacement therapy, were also lower in the SGLT2i-treated group [38]. These results point toward the fact that SGLT2i use improves outcomes in patients with cancer therapy-related cardiac dysfunction/HF [38].

While all the above-mentioned studies focus on the beneficial effects of SGLT2is in the setting of HF or cardiac dysfunction resulting from a previous antineoplastic treatment, it is intriguing to question whether Gliflozins may be useful in preventing the evolution of HF itself within this patient population. To investigate this, Chiang et al. focused on the effect of SGLT2is on the incidence of HF and mortality among patients affected by cancer and diabetes [39]. This study did not include data on the exact cause of death and treatment response; hence, it was not possible to determine whether benefits in terms of survival were due to SGLT2is’ antitumor effect or their cardiovascular benefits. Moreover, SGLT2is were associated with a significant reduction in the risk of HF hospitalization and a higher overall survival rate. The risk of severe adverse events, such as hypoglycemia and sepsis, was shown to be similar between SGLT2i recipients and non-recipients. SGLT2is have also been shown to be promising in preventing the incidence of HF in this subset of patients [39].

## 5. SGLT2is and Takotsubo Syndrome: The Two Sides of the Coin

Takotsubo syndrome is a mainly reversible condition triggered by both emotional and physical stressful conditions. The pathophysiological background is yet to be fully elucidated, but the catecholaminergic storm and beta-adrenergic receptor-stimulated apoptosis in cardiomyocytes, which are mediated by mitochondrial pathways, are among the main hypotheses [40]. It usually presents with wall motion abnormalities, such as akinesia of the apex of the LV associated with hyperkinesia of the base of the heart. In such a setting, cardiac magnetic resonance (CMR) plays a crucial role in allowing a differential diagnosis of acute MI. As of yet, there is no Takotsubo syndrome-specific treatment, which is usually quite conservative. Considering the impact that SGLT2is have on preventing cardiac remodeling and the acute structural and functional changes occurring in the setting of Takotsubo syndrome, one might question if SGLT2is may also prove beneficial within this patient population. There is, however, still little evidence in the literature on this topic, most of which focuses on single case studies [40,41].

One case report has identified a case of Takotsubo syndrome followed by ketoacidosis (associated with SGLT2is) in a patient treated with ICIs for metastatic melanoma [42].

In another case, in a patient with chronic thyroiditis, the SGLT2i triggered a thyroid crisis, and the resulting catecholamine overload resulted in Takotsubo syndrome [42]. These two examples may not necessarily imply a causative mechanism behind the use of SGLT2is in such specific conditions; nevertheless, authors cannot exclude that Gliflozins may, in some way, play a role in the metabolic disturbance leading to a Takotsubo syndrome [43].

However, data on this behalf remain controversial. In another study, rats were injected with isoprenaline, which induced elevated levels of oxidative stress in the left ventricle, as well as macrophage infiltration, while also promoting inflammation, remodeling, and fibrosis [44]. It also induced endothelial dysfunction in the systemic microcirculation, leading to a Takotsubo-like syndrome. In this study, Empagliflozin was shown to significantly prevent such adverse changes, therefore suggesting that Empagliflozin may act as a possible target for the treatment of Takotsubo [44].

It must be acknowledged that most of the information available on the topic comes from case studies or studies conducted on animal models; hence, further evidence is needed in order to better elucidate the effects of SGLT2is in the setting of Takotsubo syndrome.

## 6. SGLT2i Use in Valvular Disease: What We Know and Future Perspectives

### 6.1. SGLT2is and Aortic Stenosis

Valvular heart disease has an important burden on CV morbidity and mortality worldwide [45]. Among valvular heart disease, within the United States of America, aortic stenosis (AS) is present in about 5% of the population aged 65, with a prevalence that increases with advancing age [46]. In terms of hemodynamics, AS is responsible for the augmentation of the pressure overload, thus leading to compensatory hypertrophy of the left ventricle. Indeed, as established by the Laplace equation, the increase in pressure leads to a concomitant increase in the left ventricular wall thickness in order to maintain a normal afterload. AS can affect both the diastolic and systolic function of the left ventricle. Regarding the systolic function, hypertrophy can be inadequate to normalize wall stress, causing a reduced ejection fraction. In addition, the diastolic function can be altered. Diastole is divided into active relaxation and passive filling. The pressure decay following the aortic valve closure appears to be slower in patients with AS. The direct consequence of this is a reduced diastolic filling time and higher filling pressure, with the possible development of HFpEF with pulmonary congestion, which is another area of the effectiveness of the SGLT2i’s action [47].

A study by Urbano Pagan et al. investigated the use of SGLT2is in the setting of AS. While the prevalence of AS increases with age, no medical therapies have shown an impact on the natural history of AS. The study focused on evaluating the effects of Empagliflozin on cardiac remodeling and HF development in rats affected by AS. The study indeed demonstrated that Empagliflozin ameliorated cardiac remodeling, diastolic function, and oxidative stress while, at the same time, reducing HF frequency and interstitial collagen fraction in rats with AS [48].

Transcatheter aortic valve implantation (TAVI), despite ameliorating AS, carries a certain risk of undergoing rehospitalization within the first year post-implantation. HF, being the leading cause of rehospitalization and worsening heart failure, significantly impacts the mortality and quality of life of these patients [49,50].

The Dapa-TAVI trial is investigating the efficacy and safety of Dapagliflozin in a broad spectrum of frail patients who underwent TAVI to assess whether the use of Dapagliflozin in this setting may help in reducing the quite significant HF hospitalization risk [49]. This study demonstrated the role of SGLT2is in maintaining stable renal function and reducing cardiac stress biomarkers, such as NT-proBNP [49].

### 6.2. SGLT2is and Mitral Stenosis

The use of Dapagliflozin in mitral stenosis induced by rheumatic heart disease was poorly investigated, but the addition of Dapagliflozin to standard therapy provided benefits for these patients. This has been evidenced by an increase in net atrioventricular compliance, a reduced mean trans-valvular pressure gradient, and reduced NT-proBNP levels. This study was conducted on a small sample size with a short intervention period. Additionally, the fibrosis biomarkers considered, being present in the circulation, could also have been affected by other factors. Indeed, the authors highlight the need for and importance of further research on the topic [51].

### 6.3. SGLT2is and Mitral Regurgitation

Another valvulopathy scarcely explored in terms of SGLT2i use is mitral regurgitation (MR). In its compensated state, MR leads to a chronic volume overload, determining an enlargement of the atrium and the ventricle due to a remodeling of the extracellular matrix and a dissipation of collagen weave. Nevertheless, a progressive worsening of MR or contractile function or the increase in afterload can lead to a decompensated state and arrhythmias [52]. SGLT2is, in particular Dapagliflozin, have been proven to play a protective effect against atrial fibrillation, which is one of the most common consequences of mitral valve disease [53]. Moreover, the maladaptive remodeling associated with MR is characterized by myocardial fibrosis replacement, particularly in patients with mitral valve prolapse [54].

A further study has demonstrated that Dapagliflozin is capable of suppressing cardiac fibrosis and endoplasmic reticulum stress while improving hemodynamics in a rat model affected by mitral regurgitation-induced HF [55].

Currently, there are no relevant data in the literature on the use of Gliflozins in right-sided valvulopathies.

## 7. SGLT2is and Myocarditis: Clinical Applications and Promising Experimental Models

Although data are still scarce, there is substantial evidence in the literature that SGLT2is display beneficial effects in the setting of myocarditis.

For instance, Dapagliflozin had protective effects on Coxsackievirus B3-induced acute viral myocarditis. Indeed, by decreasing the level of pro-inflammatory cytokines and increasing anti-inflammatory macrophage polarization, the use of Dapagliflozin was associated with a significantly reduced severity of acute viral myocarditis, better cardiac function, and a more promising survival rate [56].

Canagliflozin was able to reduce cardiac inflammation and ameliorate cardiac function in the setting of experimental autoimmune myocarditis mice [57]. It reduced the expression of the nucleotide-binding domain, the leucin rich-containing family, pyrin domain-containing-3 (NLRP3) inflammasome complexes, and their downstream molecules (including interleukin-1beta and interleukin-18). Furthermore, Canagliflozin was associated with reductions in Th17 cell infiltration in the myocardial tissue and other pathways involved in myocardial cell apoptosis. These findings suggest that Canagliflozin may prove beneficial in treating myocarditis [57].

Empagliflozin has also been associated with beneficial effects in the setting of experimental autoimmune myocarditis in mice by inhibiting nuclear factor-kappa B (NF-kB)-dependent cardiomyocyte pyroptosis [58].

The fact that these studies have been conducted on animal models should be highlighted, hence the need to translate these promising experimental results into the clinical setting.

**Figure 2:**
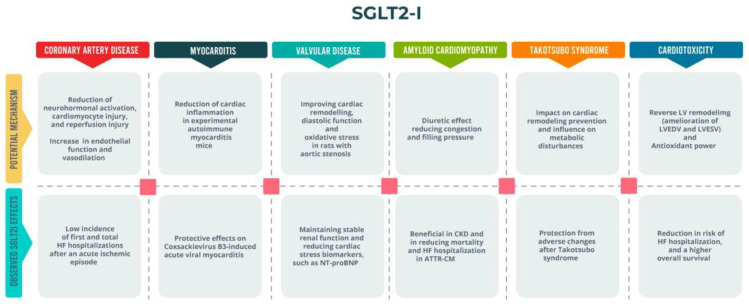
SGLT2is’ beneficial effects on different HF scenarios. ATTR-CM: Transthyretin amyloid cardiomyopathy; CKD: chronic kidney disease; HF: heart failure; LVEDV: left ventricular end-diastolic volume; LVESV: left ventricular end-systolic volume; NT-proBNP: N-terminal pro-b-type natriuretic peptide; SGLT2i: sodium-glucose cotransporter 2 inhibitor.

A summary of SGLT2i actions in these particular scenarios is shown in Figure 2.

## 8. Limitations

It must be acknowledged that most of the studies carried out on the explored topics have certain limitations. First, some specific settings are still almost entirely unexplored, and/or the study sample size is often small. Furthermore, in certain studies, the intervention period was short, hence the need for longer follow-ups. In several cases, the study populations were quite modest, and this may have had some confounding effects on the results. Additionally, in certain settings, most of the available data come from experimental models, hence the need to transcribe them in real-world clinical settings.

## 9. Conclusions

Although the guidelines do not make a clear distinction between different HF etiologies, specific subgroups of patients may particularly benefit from the pleiotropic effects of SGLT2is. The analysis of limited data suggests that HF etiology should be considered when administering SGLT2i therapy. In particular, Gliflozins may be helpful and safe in HF contexts in which cardiac remodeling and metabolic energy dysregulation are crucial. Given that this topic remains underexplored, further studies are encouraged to optimize the use of these therapies based on the underlying mechanisms of HF. The findings from this reviewed literature highlighted the following key points:

- Ischemic Heart Disease and Cardiotoxicity: primary studies demonstrate reassuring results linked to the protective metabolic and anti-inflammatory effects of SGLT2is.
- Amyloidosis and Valvular Diseases: the beneficial impact on volume overload and filling pressures appears promising, although existing data are scarce.
- Takotsubo Syndrome: results remain ambiguous, necessitating further research.
- Myocarditis: while most studies are based on animal models, the use of SGLT2is in the acute phase may be beneficial due to their known anti-inflammatory and anti-remodeling properties.
